# Sample preparation strategy for the detection of steroid-like compounds using MALDI mass spectrometry imaging: pulmonary distribution of budesonide as a case study

**DOI:** 10.1007/s00216-021-03393-6

**Published:** 2021-05-17

**Authors:** Riccardo Zecchi, Pietro Franceschi, Laura Tigli, Davide Amidani, Chiara Catozzi, Francesca Ricci, Fabrizio Salomone, Giuseppe Pieraccini, Barbara Pioselli, Valentina Mileo

**Affiliations:** 1 Mass Spectrometry Center, Department of Health Sciences, University of Florence, Viale G. Pieraccini 6, 50139 Florence, Italy; 2 Unit of Computational Biology, Research and Innovation Centre, Fondazione E. Mach, S. Michele all’Adige, 38198 Trento, Italy; 3 Preclinical R&D, Chiesi Farmaceutici, 43122 Parma, Italy

**Keywords:** Budesonide, Girard reagent, Ferulic acid, Pulmonary drug distribution, Mass spectrometry imaging, On-tissue derivatization

## Abstract

Corticosteroids as budesonide can be effective in reducing topic inflammation processes in different organs. Therapeutic use of budesonide in respiratory diseases, like asthma, chronic obstructive pulmonary disease, and allergic rhinitis is well known. However, the pulmonary distribution of budesonide is not well understood, mainly due to the difficulties in tracing the molecule in lung samples without the addition of a label. In this paper, we present a matrix-assisted laser desorption/ionization mass spectrometry imaging protocol that can be used to visualize the pulmonary distribution of budesonide administered to a surfactant-depleted adult rabbit. Considering that budesonide is not easily ionized by MALDI, we developed an on-tissue derivatization method with Girard’s reagent P followed by ferulic acid deposition as MALDI matrix. Interestingly, this sample preparation protocol results as a very effective strategy to raise the sensitivity towards not only budesonide but also other corticosteroids, allowing us to track its distribution and quantify the drug inside lung samples.

**Supplementary Information:**

The online version contains supplementary material available at 10.1007/s00216-021-03393-6.

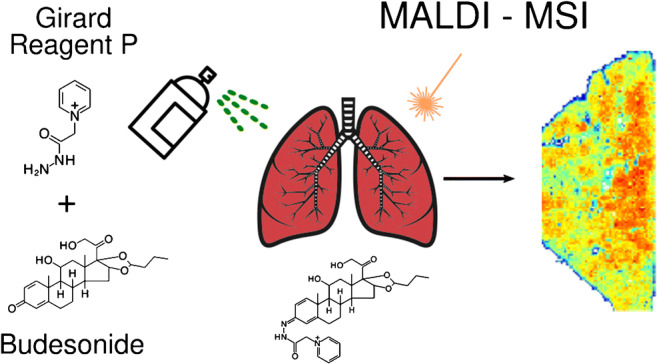

## Introduction

Budesonide (BUD) is a corticosteroid drug available since the early 1980s. It carries out its function by acting as an agonist of glucocorticoid receptors, depressing the migration of polymorphonuclear leukocytes and fibroblasts and reversing capillary permeability and lysosomal stabilization at the cellular level to prevent or control inflammation process [1, 2]. Different BUD formulations have been developed to soften inflammation that can occur at different levels of the respiratory system: nasal sprays are available to treat allergic rhinitis and nasal polyps [3, 4], and inhaled or nebulized formulations are widely used to manage long-term treatment of asthma and chronic obstructive pulmonary disease (COPD) [5–8]. Recently, its use in pulmonary diseases typical of newborns and children patients, like respiratory distress syndrome (RDS) or bronchopulmonary dysplasia (BPD), is increasing and it is under evaluation by the scientific community [9, 10]. Anti-inflammatory therapy with corticosteroids may help to reduce important comorbidities mediated by the persistence of inflammatory status that can affect the life of infants and children for many years [11, 12]. Understanding the local distribution of BUD inside the lung parenchyma is very important to fine-tune the drug dose and to optimize and compare different delivery methods, in order to reach the desired local anti-inflammatory effects. For this reason, the distribution of BUD formulations has already been investigated with molecular imaging technologies such as nano positron emission tomography (Nano/PET) and fluorescent dye imaging [13]. Mass spectrometry imaging (MSI) is a biomolecular tool that is increasingly used to monitor the distribution of endogenous and exogenous compounds in tissues [14–16]. Compared to conventional imaging techniques, MSI offers important advantages: it does not require any labelling of the molecule, it can produce high spatial resolution images, and it allows for simultaneous detection of thousands of different compounds in a single experiment. Unfortunately, not all the molecular species are readily detectable through MSI with sufficient sensitivity due to their physicochemical properties. It is well known that steroid-like compounds are difficult to detect by mass spectrometry coupled with “soft” ionization techniques (ESI or MALDI), since these molecules are not easily protonated or deprotonated. Furthermore, they are susceptible to ion suppression by more abundant molecules, such as lipids, and can suffer by isobaric overlay between the common MALDI matrix and the molecule ionization peaks. To overcome this issue, past examples in literature reported the application of a silver sputter coating of the tissue section, and subsequent LDI-MS [17, 18] or DESI-MS analysis [19]. Another strategy was to adopt a derivatization procedure that can increase ionization yield adding a charged or easily ionizable group directly on the molecule and shifting the analyte mass to higher *m/z* values where there are less matrix interferences [20–22]. Corticosteroids such as BUD have a reactive carbonyl group on C3 that can be derivatized forming oximes and hydrazones. Girard’s reagents are quaternary ammonium salts with hydrazine groups that form water-soluble hydrazones when reacting with carbonyl compounds [23]. Girard’s reagents have been commonly employed as derivatizing agents since the early 1970s for HPLC-MS detection of endogenous or exogenous steroid-like compounds, but more recently, they also proved their efficacy for on-tissue chemical derivatization (OTCD) methods suitable for MALDI-MSI analysis. In the last decade, MSI has been successfully used for the detection of steroid-like compounds in different organs such as the brain [24], the testis [25], and the adrenal gland [26], and even hard biological matrices such as cartilage [27]. Here, we developed an OTCD method using Girard’s reagent P and T (GirP, GirT) to detect BUD into lung tissues of surfactant-depleted adult rabbit RDS model, following intratracheal administration of an aqueous BUD formulation. Our findings provide an optimized MALDI-MSI approach consisting of matrix spray coating of ferulic acid after an on-tissue GirP derivatization process, able to increase ionization efficiency of derivatized towards underivatized corticosteroids by one order of magnitude. Furthermore, we succeeded in setting up a quantitative MSI method for BUD analysis, also evaluating the potential ion suppression phenomenon linked to the abundant presence of lipids in our samples [28].

## Materials and methods

### Chemicals and reagents

Ethanol, methanol, and TFA, and chemical standards of tiotropium bromide, budesonide, cortisone, testosterone, prednisone, and triamcinolone acetonide, GirP and GirT, 2,4-dinitrophenylhydrazine (DNPH), as well as MALDI matrices α-cyano-4-hydroxycinnamic acid (HCCA), 2,5-dihydroxybenzoic acid (DHB), and ferulic acid (FA) were purchased from Sigma-Aldrich Italy (Milan, Italy). Distilled water was produced by a Milli-Q (Millipore Merck, Milan, Italy) apparatus. Histology glass slides were Superfrost Plus (Thermo Scientific, Waltham, MA, USA). Meyer’s hematoxylin and eosin alcoholic solutions for tissue staining and xylene-based tissue fixing glue were purchased from Diapath (Milan, Italy). Budesonide formulation was Pulmaxan® (AstraZeneca, Cambridge, UK) while surfactant poractant alfa (Curosurf®) was provided to us by the producer (Chiesi Farmaceutici, Parma, Italy). Extemporaneous combined formulation of Budesonide and poractant alfa (surfBUD) was freshly prepared when needed by mixing 285 μL of 0.25 mg/mL Pulmaxan® with 714 μL of poractant alfa. After 2 min handshaking, we obtained an ambient temperature and time stable surfBUD formulation.

## In-solution derivatization of corticosteroids

Five different steroid compounds (cortisone, testosterone, prednisone, budesonide, and triamcinolone acetonide) were used to assess the best derivatizing agent in combination with the matrix capable to maximize the ionization yield of these compounds. Aliquots of 100 μL of an acid ethanol solution (0.2% TFA), containing 50 ng/μL of each steroid, were added with 100 μL methanol solutions containing 5 mg/mL of GirT or GirP or DNPH. Derivatization was enhanced putting the solution at 80 °C for 5 min. Each solution (0.5 μL) was then posed on a stainless steel target plate together with HCCA solution (70% acetonitrile), or DHB or FA solution (50% ethanol).

### Animals, drug administration, and tissue collecting

The experimental procedure was approved by the intramural Animal Welfare Body and the Italian Ministry of Health (Prot. n. 1300-2015-PR) and complied with the European and Italian regulations for animal care. The experiments were carried out in a 7-week-old New Zealand white adult rabbit with a body weight of 2.0 kg, obtained from Charles River Laboratories (Romans, France). Animal sedation and surgical procedures have been described by Ricci et al. Rabbit was intubated and stabilized on mechanical ventilation and underwent repeated bronchoalveolar lavages (BALs) to achieve surfactant depletion [29]. Following surfactant depletion, the animal was treated intratracheally with a bolus of 0.25 mg/kg BUD (0.25 mg/mL) as in Ricci et al. [30]. Animal was maintained in mechanical ventilation for 1 min with the following settings: inspiration fraction (Fi) O_2_ = 100%, flow = 10 L/min, respiratory rate = 40 breaths/min, positive end-expiratory pressure = 3 cmH_2_O, tidal volume (VT) targeted to 7 mL/kg (with peak inspiratory pressure not higher than 23 cmH_2_O), and inspiratory time of 0.5 s. After the observational period, the animal was killed by exsanguination. Lungs were removed and inflated with air to 20 cmH_2_O and suspended in a Dewar flask containing liquid nitrogen in the bottom. The lung froze above the liquid nitrogen in the cold environment in about 10 min. The lungs were stored at − 80 °C until analysis.

### Preparation of homogenized lung tissues

Tissues from untreated adult rabbit samples, obtained from Charles River Laboratories (Romans, France), were thawed and the parenchymal tissues were dissected from main bronchus and bronchioles using a sterile lancet and lab scissors. Parenchymal pieces were placed in a T10 basic Ultra-Turrax (IKA Laboratory, Staufen, Germany) mixer for 3 min to obtain a uniform homogenate without adding water. Aliquots of homogenate lung tissue were put in pre-weighted 500-μL Eppendorf tubes. Some of these aliquots were spiked and mixed again, then frozen in a − 80 °C freezer. Two homogenized tissue aliquots were spiked with 20% (w/w) or 10% (w/w) of poractant alfa, obtaining mixtures with surfactant concentration of 8 mg/g and 4 mg/g, respectively. Other two aliquots were spiked before freezing with 20% (w/w) or 10% (w/w) of surfBUD, obtaining mixtures with BUD concentration of 14.2 μg/g and 7.1 μg/g, respectively. The last two aliquots were spiked with amounts of Pulmaxan® to obtain the same concentration of BUD reported for the ones spiked with surfBUD (14.2 μg/g and 7.1 μg/g).

### Tissue sectioning and staining

The cryostat CM1860UV (Leica Microsystems, Wetzlar, Germany) was used for sectioning the homogenized control tissue and the animal lung lobes as fresh-frozen samples, cut among frontal plane at middle height. We collected the tissue sections at 20 μm thickness, thaw-mounted them over the glass slides and allowed to dry in a vacuum desiccator (15 min). In the end, every glass slide accommodated one sample section and two control homogenized sections spiked with 2.5 ng and 10 ng of budesonide standard. Glass slides used for MSI analysis were subsequently washed for matrix removal with ethanol and processed with hematoxylin and eosin standard protocol for tissue staining and subsequent dehydration and fixation.

### On-tissue chemical derivatization

A solution of 10 mg/mL of GirP in 50% methanol, containing 0.2% TFA and the internal standard triamcinolone acetonide at 2 μg/mL concentration, was distributed for OTCD using the iMatrixSpray device (Tardo GmbH, Subingen, Switzerland). Samples were placed onto the heated plate of the device at 80 °C and covered by 30 layers of GirP solution (density of 0.5 μL/cm^2^, nitrogen flow at 120 bars, needle distance of 6 cm). Total spray time was 40 min, but we maintained the samples over the heated plate for additional 30 min to be sure that derivatization process fully completed. After this procedure, all the glass slides were put in a vacuum desiccator for 15 min.

### Matrix application

Matrix (FA, 15 mg/mL in 50% ethanol) was applied in 50 cycles using iMatrixSpray device (density of 0.7 μL/cm^2^, nitrogen flow at 120 bar, needle distance of 6 cm, heated beads at 60 °C). After matrix coating, samples were finally dried in a vacuum desiccator for 15 min.

### Quantitative method development

A dilution series of budesonide was prepared in 50% ethanol at the following concentration: 0.2, 2, 5, 20, and 40 ng/μL; 2.5 ng/μL of tiotropium standard was added in every solution for data analysis purposes. Drops of 500 nL of each solution were placed onto homogenized tissue sections. In order to investigate differential lipid content of our samples, calibration curve series were prepared on untreated lung homogenate slices, as well as on the homogenate previously spiked with 4 mg/g and 8 mg/g of poractant alfa. All the calibration curve series were prepared in four replicates in order to allow a better statistic.

### MALDI-MSI acquisition

A MALDI-LTQ-Orbitrap XL mass spectrometer (Thermo Fisher, San José, CA, USA) equipped with a 60-Hz nitrogen laser emitting at 337.1 nm was used to acquire MALDI-MS and MALDI-MSI spectra. MSI data were performed using a raster size of 400 × 400 μm in the mass range from *m/z* 185 to 650 in positive ion mode, using 60,000 resolving power (at 400 *m/z*) for the FT analyzer. For every position, we accumulated 3 microscans of 10 laser shots each, with 8 μJ fixed laser energy. Internal lock mass was set for peak at *m/z* 195.0657, representing the molecular ion of FA. All the data were acquired as centroided and then converted with “RAW to imzML” software (Giessen University, Giessen, Germany).

### Data analysis

All data analyses were performed in R [31] relying on the package “tidyverse” [32] for data management and visualization. The ion images (tolerance = 0.005 Da) for the compounds of interest were extracted from the raw experimental data by using the pyimzML parser [33] through the reticulate [34] R interface. All signals were log10 transformed to compensate for the expected non-normal distribution of count data. The different tissue sections/areas were identified by a segmentation approach (package EBImage [35] and raster [36]) on the endogenous heme B [M + H]^+^ ion signal (*m/z* = 616.177) and manually annotated. The budesonide-GirP [M + H]^+^ ion signal (*m/z* = 564.308) was normalized by the triamcinolone acetonide-GirP [M + H]^+^ ion signal (*m/z* = 568.283), used as internal standard, in order to take into account the variability from pixel to pixel. In the case of the calibration curves, the pixels belonging to the spot areas were automatically identified by using the tiotropium [M + H]^+^ ion signal (*m/z* = 392.098) with a manually adjusted threshold of 5.6.

## Results and discussion

### In-solution derivatization of corticosteroids

**Fig. 1:**
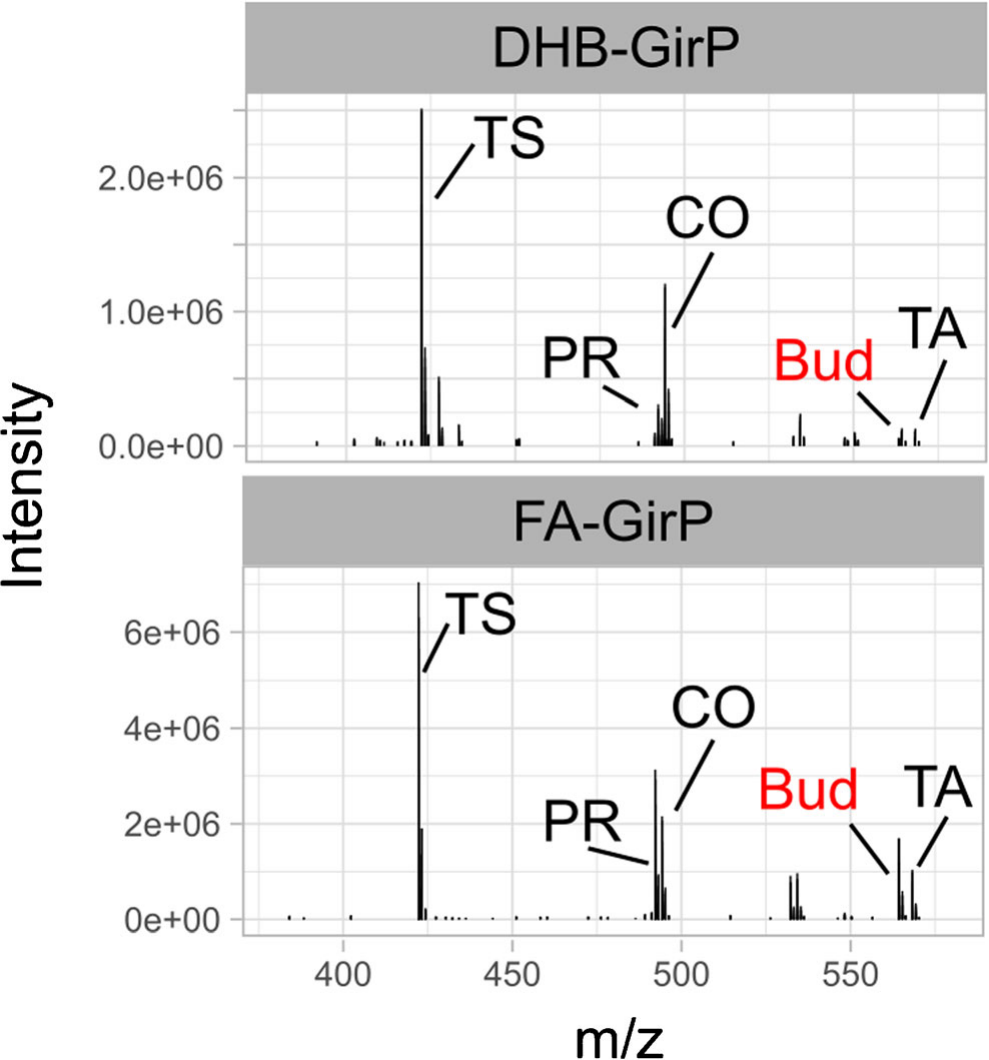
Comparison of in-solution derivatized corticosteroids. MALDI-FT full scan mass spectra of six Girard’s P derivatized corticosteroids. Compound mixture was then spotted onto MALDI plate and analyzed with 2,5-dihydroxybenzoic acid (DHB) or ferulic acid (FA). Testosterone (TS), prednisone (PR), cortisone (CO), budesonide (BUD), and triamcinolone acetonide (TA)

Low sensitivity is often one of the great drawbacks of MSI technology and many efforts need to be spent when analytes show poor ionization yield. Because of its hydrophobic properties, BUD as other corticosteroids required a chemical derivatization process in order to be revealed with ESI or MALDI ionization. For these reasons, detecting and mapping the distribution of BUD with MALDI-MSI requires a careful optimization of sample preparation method. With the aim of understanding which was the best combination between different derivatizing agents and matrices, an initial survey of the response of standard steroid molecules spotted on a MALDI target plate was performed. Three hydrazine compounds already reported in literature (GirT, GirP, and DNPH) [37, 38] were chosen as derivatizing candidates and tested for in-solution derivatization of budesonide, testosterone, prednisone, cortisone, and triamcinolone acetonide. Every derivatizing agent was tested in combination with HCCA, DHB, and FA matrix. DNPH showed very poor results in terms of sensitivity compared to Girard’s reagents and, therefore, we decided to discard this compound after the very first trials. Results with GirT and GirP (Fig. 1, Supplementary Information (ESM) Fig. S1) highlighted that both reagents worked with comparable efficiency on all the steroids, allowing the detection of the protonated molecular ions of derivatized steroids. It was also evident that combination with FA matrix enhanced the detection of all the compounds. With this matrix, the peak intensity of all the investigated steroids was at least five times higher, compared to the results obtained with DHB and HCCA matrices. Testosterone and cortisone showed a good sensitivity in all the conditions. On the other hand, prednisone, BUD, and triamcinolone acetonide ions were at low intensity in the spectrum (5% of the highest peak height) when using HCCA or DHB, but FA matrix was responsible of a great increase on the yield of the protonated ions of these derivatized corticosteroids resulting in better signal-to-noise ratio. In particular, GirP derivatization gave a slightly higher sensitivity than GirT, and showed smaller crystals and more uniform coverage when sprayed over the tissue surface; for these reasons, GirP was preferred for our MSI analyses. BUD-GirP derivative structure was confirmed by high-resolution MS/MS experiment and compared with mass spectrum present in literature [39] (ESM Fig. S2).

### Method development for on-tissue chemical derivatization

**Fig. 2:**
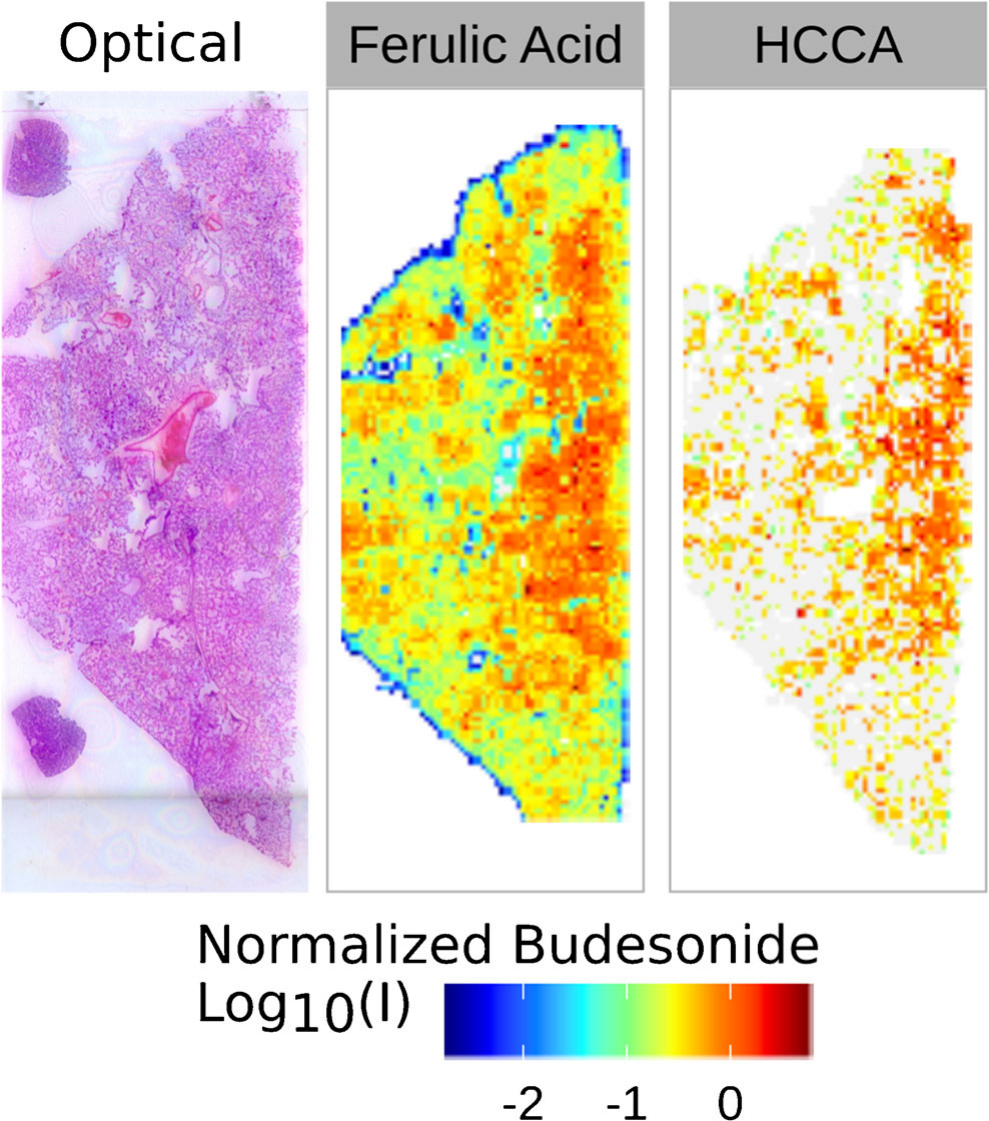
MSI analysis with different matrices. Comparison of the response of the budesonide ion (*m/z* 564.308) on consecutive lung sections when FA and HCCA are used as MALDI matrix in MSI experiments

A comparative MSI experiment employing GirP with HCCA or FA was repeated on a BUD-treated lung tissue sample, in order to assess the validity of our findings also for the OTCD. Figure 2 clearly shows that FA was effective in raising the detection efficiency of derivatized BUD protonated ion when the drug was administered at therapeutic dosage. On the contrary, HCCA matrix produced poorer results, close to the analytical limit of detection. In order to obtain these results, some efforts were needed to optimize the OTCD method. It is known that Girard’s reagents need a protic solvent with a moderate acidic environment in order to re-act with steroid-like compounds, but the optimal conditions have to be found by trial and error. In our case, GirP methanol solution with 0.2% TFA assured the delivery of an even layer of derivatizing agent, able to correctly crystallize after a few seconds over the slide. A key parameter, when OTCD is performed, is the time of derivatization. For this reason, a preliminary on-tissue time course experiment was performed. Based on literature [25, 27], we firstly used a humidity chamber (80% relative humidity, 38 °C) leaving the samples inside for 60 or 120 min after derivatizing agent spray procedure. Delocalization of analytes was massive using this device; in addition, data analysis showed us that the efficiency of derivatization was not strongly dependent on time, being already quantitative after 60 min. In order to avoid the excess of humidity and reduce delocalization, we tested another derivatization protocol that consists in leaving the sample on the heated plate (80 °C) of the iMatrixSpray device. In this case, several reaction times (0 min, 15 min, 30 min, 60 min, 90 min, 120 min) were tested, in addition to the time needed for GirP spray coating. Results showed in ESM Fig. S3 indicated that for times up to 30 min, the reaction takes place with maximum intensity; for reaction times longer than 60 min, the BUD ion signals tend to slightly decrease, indicating a possible degradation of analytes mediated by heat stress. The use of a heated plate (80 °C) under the glass slides was effective in reducing the humidity on the tissue surface, limiting undesired analyte delocalization during spray coating and extra reaction time (ESM Fig. S4). For these reasons, 30 min of extra reaction time over the heated plate was considered the appropriate time to conduct all the experiments.

### Optimization of on-tissue quantification method

**Fig. 3:**
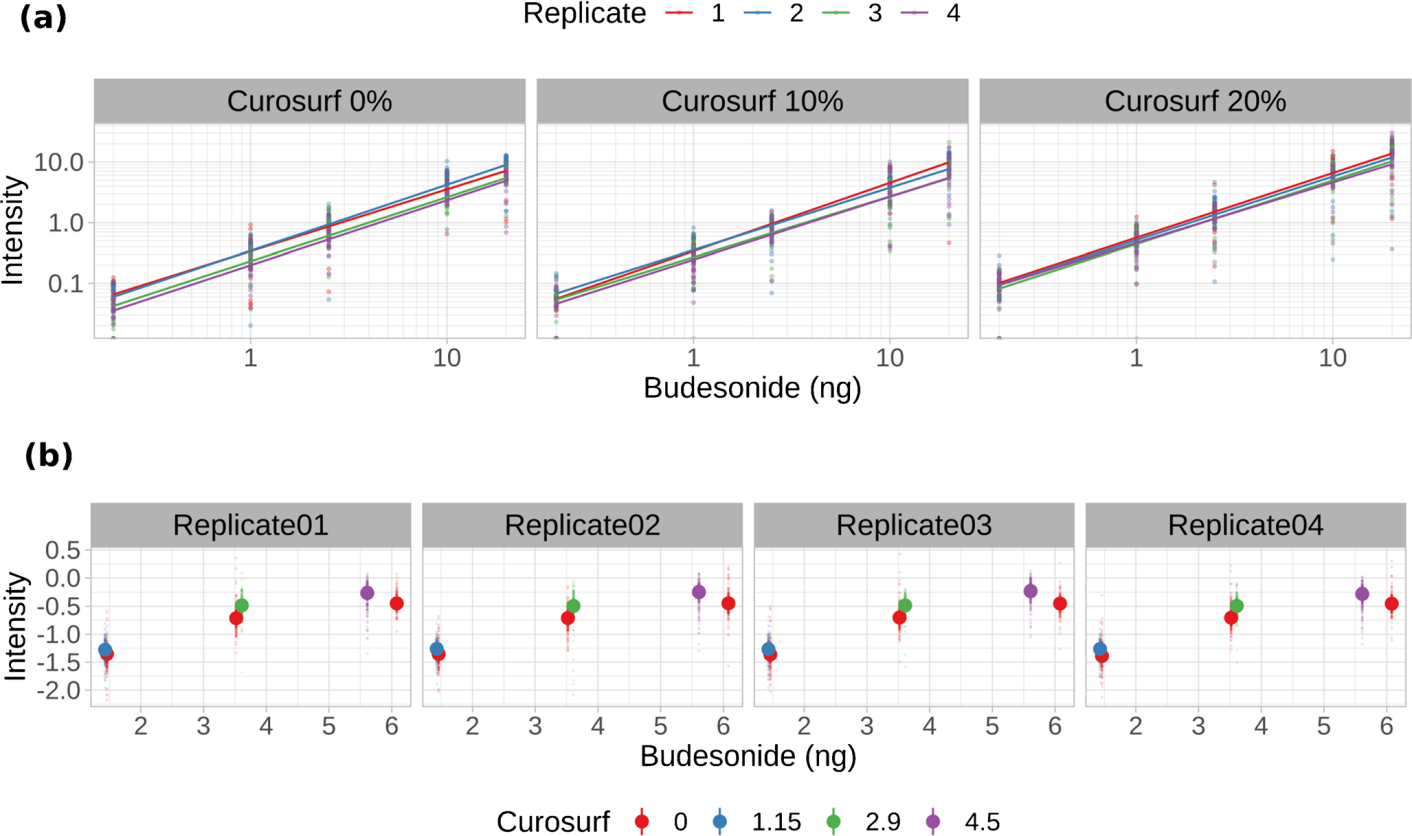
Investigation of linearity and reproducibility of the on-tissue calibration curves. **a** Linearity of the response of dilution series of budesonide spotted on normal control homogenate tissue slices in the presence of a different amount of poractant alfa. **b** Reproducibility of the response on tissue homogenates spiked either with BUD alone or with surfBUD combination

It has been shown that when a dilution series is applied on tissue, a standard curve for compound quantification can be obtained [40]. Furthermore, the addition of an internal standard, uniformly present all over the tissue surface, improves scan-to-scan reproducibility and linearity of the calibration curve [41]. Quantification strategies using an internal standard, an isotopologue or a chemical analogue of the target analyte, have been successfully applied to quantify drug in tissues. We decided to employ triamcinolone acetonide as internal standard. This choice was based on the evidence that the two molecules have strong structural similarities, a very low mass difference (4 Da) and almost equal response in MALDI ionization as GirP derivatives, as observed during the tests for in-solution derivatization. Adding the optimized amount of internal standard to the GirP solution prior to on-tissue derivatization allowed us to uniformly spray it over the samples already in its derivatized form [42, 43]. In the specific case of lung MSI, another issue that needs a special consideration is the ion suppression phenomenon potentially induced by tissue lipid content: lung tissues constitutively have an elevated lipid content deriving both from biological cell membranes and from the endogenous pulmonary surfactant present on the surface of alveoli. The high lipid content present in the samples can cause erroneous quantifications if ion suppression is not taken into consideration when calibration curves are built on tissue sections [44]. To assess the impact of different concentration of phospholipids on the reproducibility of the calibration curves, four replicates of dilution series of BUD were spotted on normal control homogenate tissue slices as well as on control tissues previously spiked with 4 mg/g and 8 mg/g of poractant alfa. As shown in Fig. 3a, the calibration curves are not strongly affected by an increased concentration of phospholipids. A linear modeling approach was applied to quantify the effect of poractant alfa addition on the intercept and the slope of the calibration curves. The model showed an excellent level of fit (*R*^2^ = 0.88) and indicated that an additional presence of phospholipids had a significant effect (~10% for the higher poractant alfa dose) on the intercept of the calibration curves, without affecting their slopes. It is well known that spotting compounds over a tissue surface is not equal to have them embedded in the tissues [45]; for this reason, further confirmative experiments were performed on tissue homogenates spiked either with BUD alone or with surfBUD combination. These spiked homogenate series were built keeping constant BUD concentration at three levels: 0 μg/g, 7.1 μg/g, and 14.2 μg/g. The results are shown in Fig. 3b. The plots show the median and the interquartile range of the normalized budesonide signal in homogenates enriched with increasing levels of poractant alfa. The figure confirms the limited effect of the phospholipid tissue content on the overall response of budesonide.

### Lung distribution of budesonide formulations

**Fig. 4:**
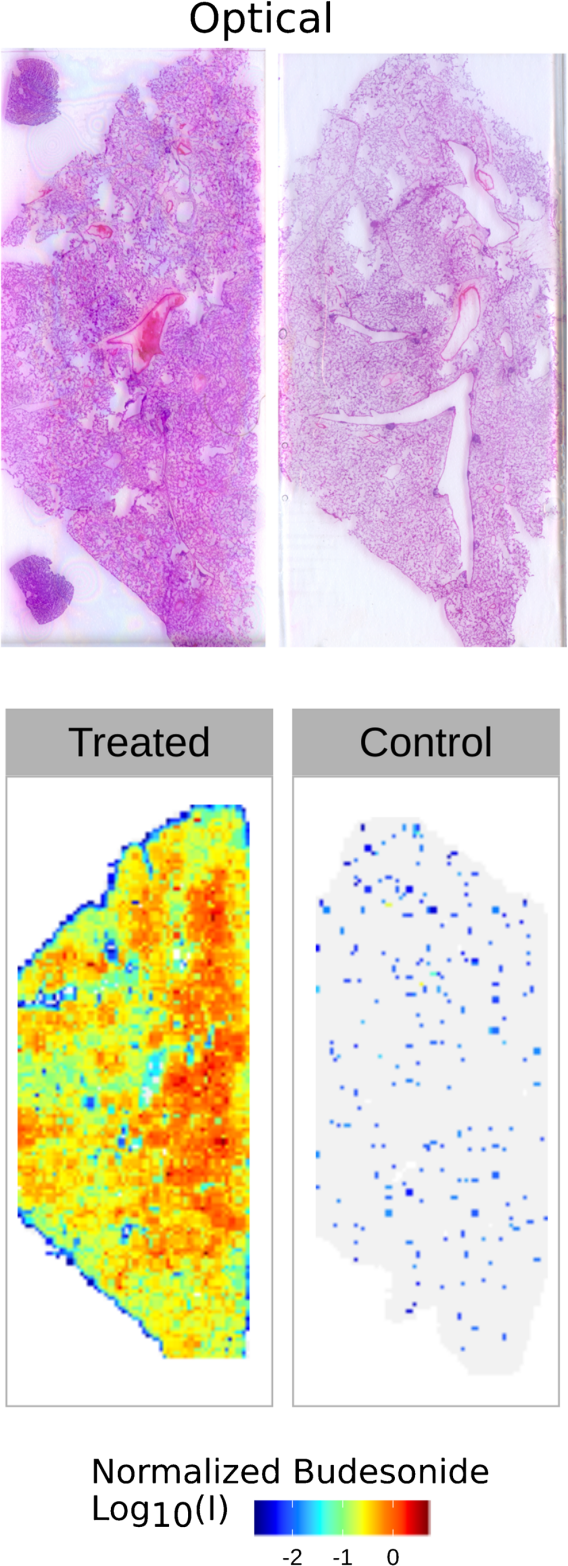
MSI analysis on rabbit lung sections. Bottom panels represent the distribution maps of budesonide ion (*m/z* 564.308) on a frontal lung section on treated (left) and control rabbits (right). Corresponding H&E-stained tissue section optical scans are represented in top panels

Although nowadays MSI can reach a spatial resolution of 10 μm or less, the distribution of BUD was investigated at 400 μm spatial resolution, due to the large dimension of the lung sections and the relatively low duty cycle of our instrumental method set-up (2.5 s per FT scan, repeated for approx. 8000 pixels consists in 6–7 h analysis time). The ion maps for the images reported in Fig. 4 were obtained normalizing the budesonide-GirP [M + H]^+^ ion against the triamcinolone acetonide-GirP [M + H]^+^ ion. Selectivity of our MSI method is confirmed by comparison with the untreated sample. Sensitivity towards target molecule is assured by at least three orders of magnitude in peak intensity. The setup of an appropriate and repeatable protocol for MSI analysis, together with a quantification method that takes into consideration differential ion suppression due to lipids, sets the basis to correctly compare different BUD administration protocols and formulations in future studies.

## Conclusions

This study reports an innovative method to analyze steroid compounds through MALDI-MSI. Girard’s reagents for on-tissue steroid derivatization have been successfully employed for testosterone, corticosterone, and triamcinolone acetonide detection, but never for budesonide. A strong innovation on sample preparation protocol is here reported, demonstrating the effectiveness of ferulic acid as MALDI matrix able to increase ionization yield up to five times compared to HCCA and DHB. Our work provides an important step for further studies examining the distribution of budesonide, a widely used drug to reduce inflammation in different organs. The increasing interest towards combined formulations of budesonide and surfactant for treating neonatal pulmonary diseases and the need of mapping its distribution confirm the benefit of the application of MSI in this field and enforce its application for further studies.

## Supplementary information

ESM 1 (DOCX 758 kb)
