# Supplementary material for: Sample preparation strategy for the detection of steroid-like compounds using MALDI mass spectrometry imaging: pulmonary distribution of budesonide as a case study
Source: Anal Bioanal Chem. 2021 May 17;413(16):4363–71. doi: 10.1007/s00216-021-03393-6 (PMC8222037; doi:10.1007/s00216-021-03393-6)
Supplement: Supplementary file 1 — (DOCX 758 kb) [file 216_2021_3393_MOESM1_ESM.docx]

Supplementary Information


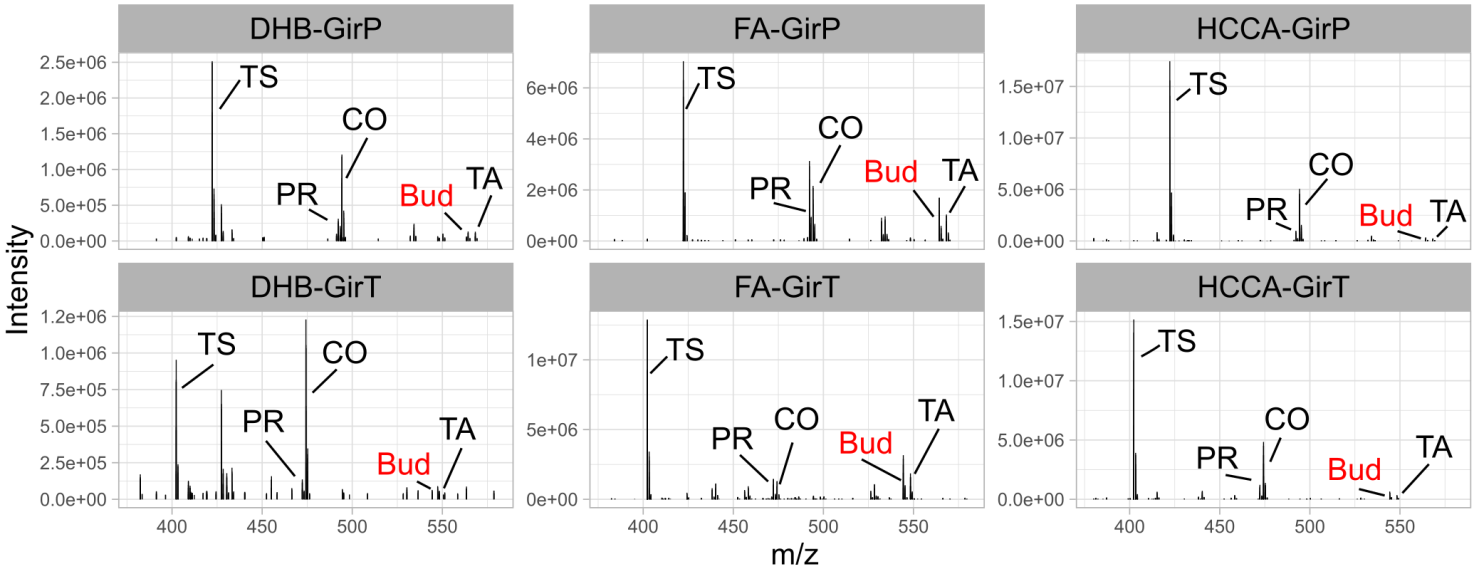


Fig. S1. Comparison of in-solution derivatized corticosteroids. MALDI-FT full scan mass spectra of six Girard’s T and P derivatized corticosteroids. Compounds mixture was then spotted onto MALDI plate and analyzed with different MALDI matrices: 2,5-dihydroxybenzoic acid (DHB), ferulic acid (FA), α-cyano-4-hydroxycinnamic acid (HCCA). Testosterone (TS), prednisone (PR), cortisone (CO), budesonide (BUD) and triamcinolone acetonide (TA).


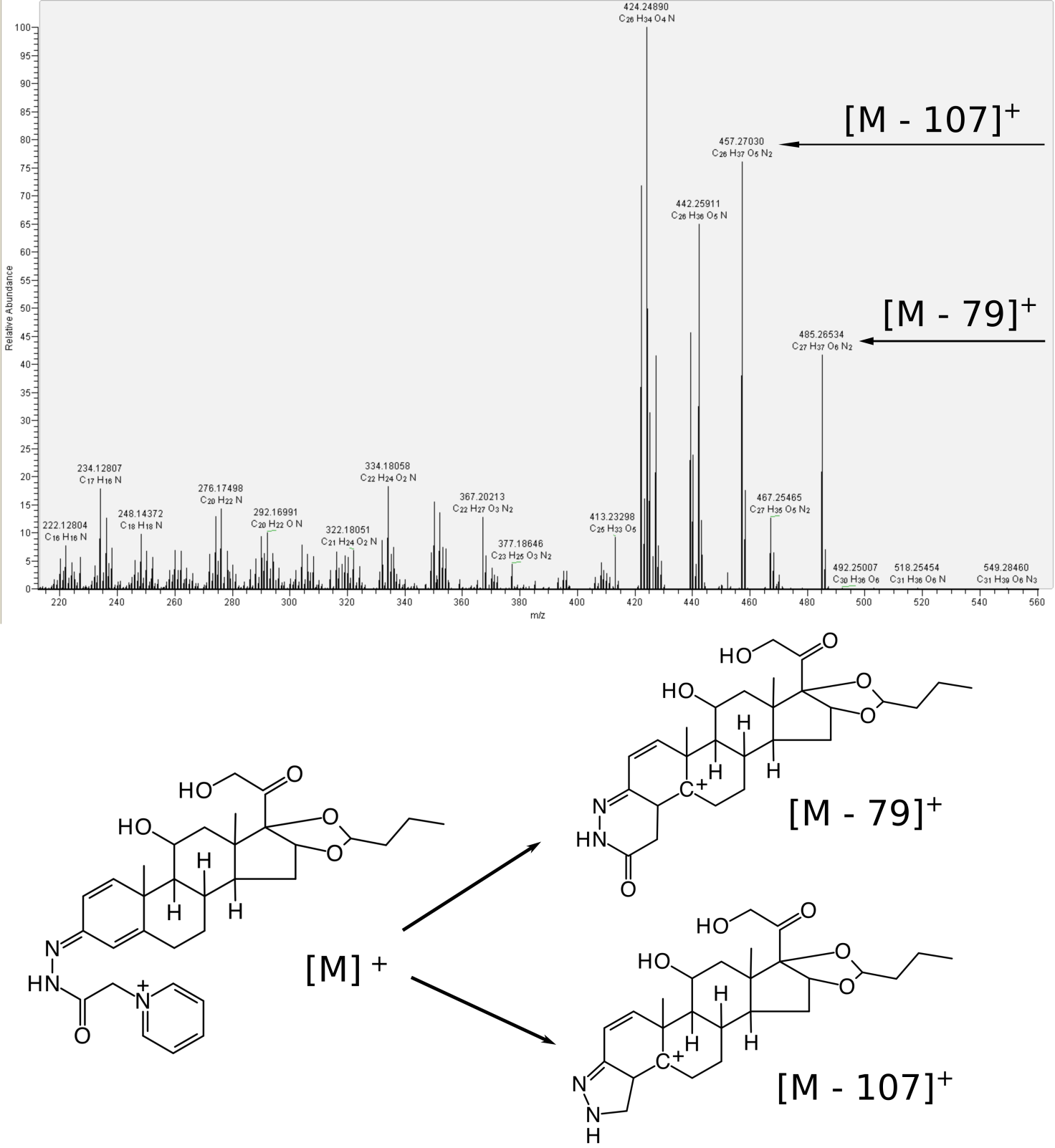


Fig. S2. MS/MS spectrum of budesonide-GirP derivative. MS/MS spectrum taken at 60000 mass resolving power shows characteristic peaks of budesonide-GirP derivative product ions at 485.285 m/z and 457.270 m/z representing the structures [M – 79]^+^, [M – 107]^+^ elucidated below the mass spectrum.


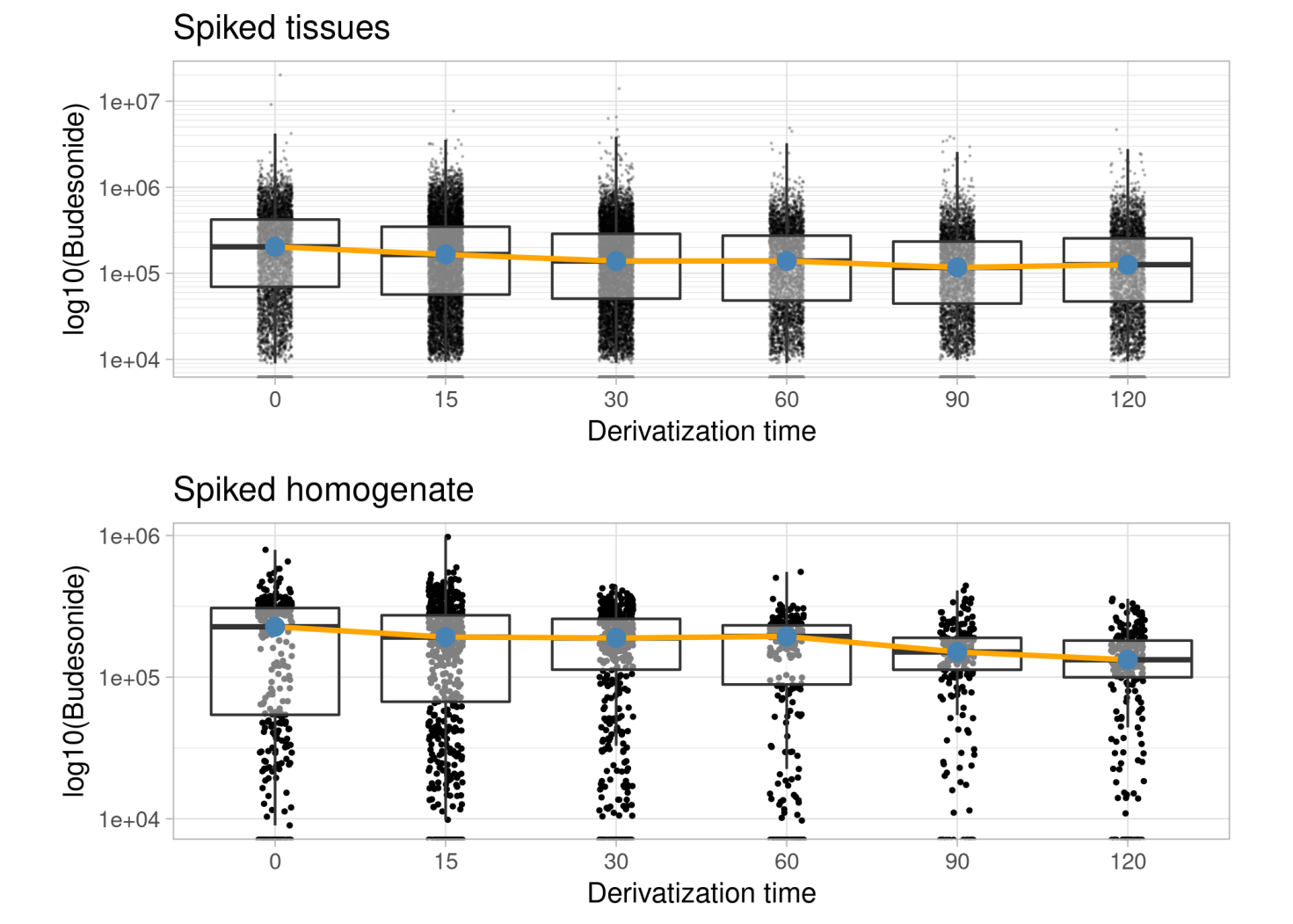


**Fig. S3. Derivatization time course experiment.** Investigation of the effect of derivatization time on the budesonide signal on Spiked Tissues and Spiked homogenates. The blue dots highlight the position of the median intensity. Orange lines highlights the trend in the overall response.


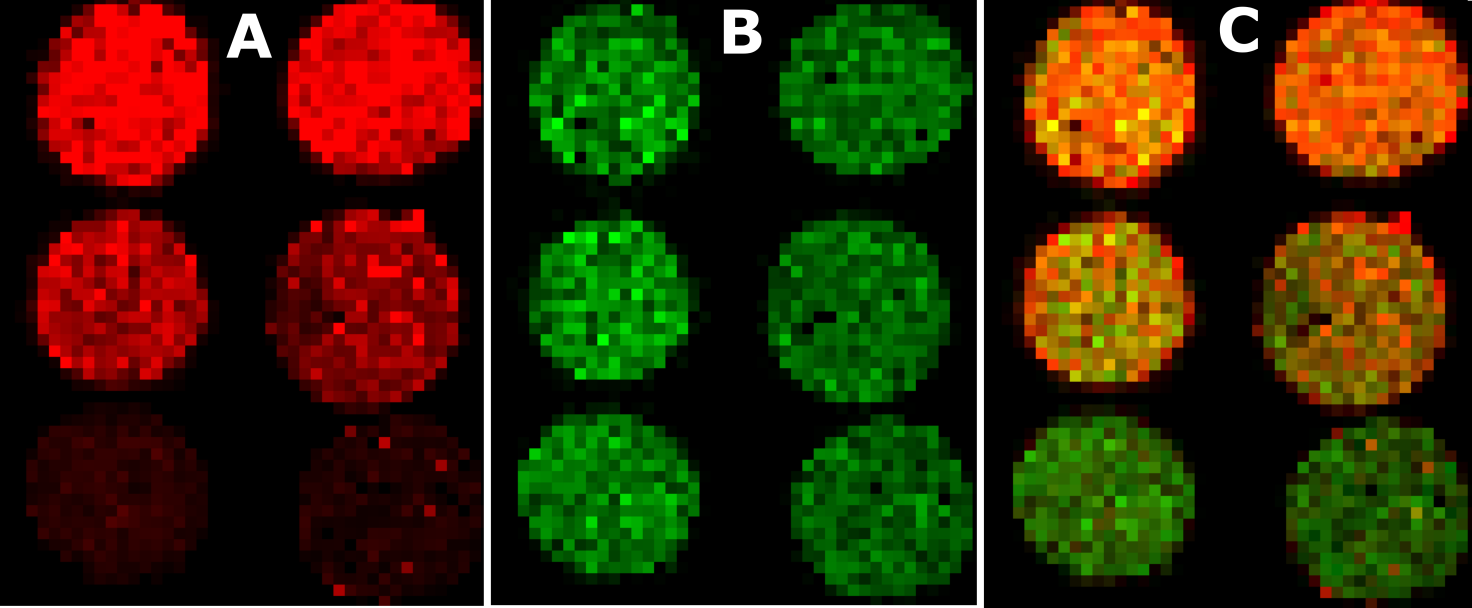


**Fig. S4. Investigation of analyte diffusion on homogenized tissue samples spiked with budesonide.** Comparison of budesonide-GirP extracted ion image at m/z 564.308 (Panel A) with Heme B extracted ion image at 616.177 m/z (Panel B). Combined ion image (Panel C) highlights absence of analyte diffusion at chosen spatial resolution of 400 x 400 µm.
